# Temporal Analyses of the Response of Intervertebral Disc Cells and Mesenchymal Stem Cells to Nutrient Deprivation

**DOI:** 10.1155/2016/5415901

**Published:** 2016-02-10

**Authors:** Sarah A. Turner, Karina T. Wright, Philip N. Jones, Birender Balain, Sally Roberts

**Affiliations:** ^1^Spinal Studies, Robert Jones and Agnes Hunt Orthopaedic Hospital NHS Foundation Trust, Oswestry, Shropshire SY10 7AG, UK; ^2^ISTM, Keele University, Keele, Staffordshire ST5 5BG, UK; ^3^Spinal Disorders, Robert Jones and Agnes Hunt Orthopaedic Hospital NHS Foundation Trust, Oswestry, Shropshire SY10 7AG, UK

## Abstract

Much emphasis has been placed recently on the repair of degenerate discs using implanted cells, such as disc cells or bone marrow derived mesenchymal stem cells (MSCs). This study examines the temporal response of bovine and human nucleus pulposus (NP) cells and MSCs cultured in monolayer following exposure to altered levels of glucose (0, 3.15, and 4.5 g/L) and foetal bovine serum (0, 10, and 20%) using an automated time-lapse imaging system. NP cells were also exposed to the cell death inducers, hydrogen peroxide and staurosporine, in comparison to serum starvation. We have demonstrated that human NP cells show an initial “shock” response to reduced nutrition (glucose). However, as time progresses, NP cells supplemented with serum recover with minimal evidence of cell death. Human NP cells show no evidence of proliferation in response to nutrient supplementation, whereas MSCs showed greater response to increased nutrition. When specifically inducing NP cell death with hydrogen peroxide and staurosporine, as expected, the cell number declined. These results support the concept that implanted NP cells or MSCs may be capable of survival in the nutrient-poor environment of the degenerate human disc, which has important clinical implications for the development of IVD cell therapies.

## 1. Introduction

Degeneration of the intervertebral disc (IVD), which is often associated with low back pain, is attributed to altered cell activity and a reduced number of healthy functioning cells within the extracellular matrix [1, 2]. Many factors have been reported which impact cell viability and metabolism, including mechanical loading, osmolarity, and reduced nutrient levels [3–5]. This reduction in nutrient availability such as levels of glucose, for example, to cells in the degenerate IVD is thought to occur via occlusion of one of the main nutrient pathways, through the cartilaginous and vertebral endplate due to increased calcification [6–8]. Degeneration appears to commence and be predominant in the central region of the disc, the nucleus pulposus (NP), and within the first decade of life in humans [9].

Autologous chondrocyte implantation (ACI) has been in use in the clinic for several years for the treatment of cartilage defects and early osteoarthritis [10–12]. Studies have demonstrated that the implantation of autologous disc cells into IVD defects may have similar potential for some degree of tissue regeneration, as shown in animal models of degenerate disc disease [13, 14]. IVD repair following the implantation of cultured bone marrow derived mesenchymal stem cells (MSCs) in animal studies has also been reported, though the mode of action remains unclear (i.e., whether the cells act via a paracrine effect by direct synthesis of matrix). Certainly implanted MSCs have been retained in the IVD tissue and remained viable for up to 6 months [14–18]. Following the reported successes in animal studies, these techniques have started to be translated into clinical trials in humans, where autologous IVD cells or MSCs have been implanted for tissue regeneration [19]. The most promising reported clinical study of cell therapy for disc repair describes a reduction in pain and return of IVD hydration to more normal levels in some patients, following autologous IVD cell implantation [20], although long-term follow-up studies are lacking.

The development of cell transplantation techniques for the repair of degenerate IVD tissue clearly has some exciting potential. However, the nutrient-poor environment of degenerate IVD tissue could be detrimental to the implanted cells. For example, following implantation, cell populations may not receive the nutrient support required for any reparative function, or even for their survival. In addition, there is some suggestion that MSCs may be less suitable for implantation into degenerate IVD tissue as they are less adapted to survival in nutrient-poor environments in comparison to native IVD cells [21]. Hence, a suitable cell source needs to be identified which will be able to survive and repair the damaged tissue under nutrient-poor conditions.

In this study, we assessed the response of IVD cells and MSCs to reduced nutrient levels* in vitro* using a temporal live cell imaging and analysis system. In addition to identifying a cell population that can survive nutrient deprivation, we aimed to investigate the environmental insults required to induce apoptosis, which is reported in cells throughout degenerate IVD tissue [22–25]. In 2009, a technique of automated microscopic time-lapse imaging technology was reported for tracking rabbit notochordal and chondrocyte-like cells in culture [26]. We report here on a similar method for longitudinal automated quantitative analysis of cell cultures which we have used to examine the response of NP cells (both human and bovine) and human MSCs to altered nutrient levels, utilising time-lapse imaging to quantify cell size and viability, before further assessing induction of cell death.

## 2. Materials and Methods

### 2.1. Bovine and Human NP and MSC Source

NP cells were isolated from bovine caudal intervertebral discs obtained within 2 hours of death from a local abattoir. In addition, with local research ethical committee approval and informed consent, human NP cells were isolated from five patients (four males and one female, aged 35 ± 7.5 years) undergoing routine lumbar vertebral fusion procedures, for the treatment of low back pain. MSCs (from bone marrow aspirates or iliac crest bone chip) were also obtained from two of these patients (both males, aged 36 and 40 years), plus a further female patient (aged 56 years).

### 2.2. Bovine NP Cell Isolation and Culture

NP cells were isolated from the caudal discs of 3 adult cows. The central NP tissue was dissected out and the three upper discs were pooled from each animal. Tissue was finely minced and digested overnight with collagenase XI (0.8 mg/mL (≥960 CDU/mL)) in DMEM/F12 medium (both from Sigma-Aldrich, St. Louis, USA) at 37°C. Cells were then maintained in culture at 5000 cells/cm^2^ in DMEM/F12 culture medium (Life Technologies, Paisley, UK) routinely supplemented with 10% (v/v) foetal bovine serum (FBS) (PAA, Yeovil, UK), 50 *μ*g/mL ascorbic acid (Sigma-Aldrich), 0.5% (v/v) gentamicin (Life Technologies), and 0.05% fungizone (Life Technologies), at 37°C in a humidified atmosphere of 5% CO_2_. Cells were passaged as standard by trypsinisation upon reaching 80% confluence and used at passage 4 or 5.

### 2.3. Human NP Cell Isolation and Culture

Freshly excised human NP tissue (identified macroscopically and confirmed microscopically) was dissected from the IVD tissue obtained at surgery. Cells were isolated and maintained in the same way as the bovine NP cells and used at passages 2–4.

### 2.4. Human MSC Isolation and Culture

Iliac crest bone marrow aspirates or iliac crest bone chip washouts were centrifuged over Lymphoprep (Fresenius Kabi Ltd., Runcorn, UK) for 20 minutes at 900 g to isolate the mononuclear cells via a density gradient. Harvested mononuclear cells were then centrifuged for 10 minutes at 750 g and a cell count was performed. The mononuclear cells were plated at 20 × 10^6^ cells per 75 cm^2^ tissue culture flask, in DMEM/F12 supplemented with 20% (v/v) FBS (Life Technologies) and 1% (v/v) of 10,000 units/ml penicillin and 10,000 mg/ml streptomycin (Life Technologies). After 24 hours, nonadherent cells were removed and the remaining adherent cells were routinely cultured at 5000 cells/cm^2^ in DMEM/F12, supplemented with 10% (v/v) FBS (Life Technologies) and 1% (v/v) penicillin and streptomycin (Life Technologies). Cells were passaged as standard by trypsinisation upon reaching 80% confluence.

### 2.5. Nutrition Response Assays

Human and bovine NP cells and human MSCs were seeded into 24-well tissue culture plates at 5,000 or 10,000 cells/cm^2^ and maintained at 37°C in a humidified atmosphere of 5% CO_2_, overnight, to allow cell adherence. Immediately prior to imaging, routine culture medium was removed from each well and replaced with “assay” medium in triplicate. Eight assay medium types were made using combinations of 0%, 10%, and 20% (v/v) FBS as well as no glucose, moderate (standard) glucose (3.15 g/L), and high glucose (4.5 g/L) medium (Life Technologies).

### 2.6. Induction of Bovine NP Cell Death and Apoptosis

Bovine NP cells were seeded into tissue culture plates and allowed to adhere overnight. The cells were then monitored for 18 hours following treatment with one of three classical cell death inducers: either introduction of culture medium (i) supplemented with 750 *μ*M hydrogen peroxide (Sigma-Aldrich) or (ii) 2 *μ*M staurosporine (Sigma-Aldrich) or a more physiological insult of serum deprivation (with 0% FBS). A control culture was included of cells grown in standard culture conditions of culture medium containing 10% FBS culture medium and 3.15 g/L glucose.

### 2.7. Automated Longitudinal Image Capture and Quantitation

Cellular responses to changes in the glucose or FBS content in medium and cell death induction were monitored by the collection of a series of digital images using the Cell-IQ Analyser (V2) automated image capture system (CM Technologies Oy, Tampere, Finland). In this closed system, cells were maintained in an optimised environment of 37°C and premixed 5% CO_2_ with air (BOC Gases, Guildford, UK). The premixed gas was delivered directly to the culture plates through a patented perfusion lid, designed to allow the gas to be delivered under a stop-flow regime, providing an optimal uniform concentration of gas across the entire plate.

**Figure 1:**
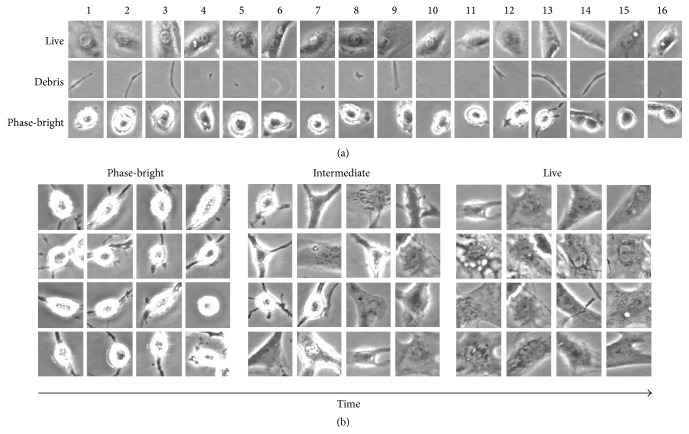
Sample library generated using the Cell-IQ® Analyser software. (a) Samples within the library are divided into three categories: live, phase-bright, and debris. Examples are added to the library to teach the software into which category a cell should fall. (b) Transition of human nucleus pulposus cells from a phase-bright category to a live, fibroblast category, over 48 hours.

Following the appropriate medium application, at least one region of interest (approximately 975 × 730 *μ*m) was selected per well and phase contrast images were collected every 20–30 minutes for 48 hours. Images were captured using a 20 *μ*m Z-stack. By creating a library of sample images, the Cell-IQ Analyser software was taught to identify cells as live or phase-bright and also to dismiss debris within the region of interest (Figure 1(a)). The total cell area, using threshold gating analyses at each individual time point (for each image), was also determined, in each culture condition tested. This provided the mean area coverage (total cells area/cell number). Temporal trends for live cell number (the sum of both live and phase-bright cell classes) and area were compared for each culture condition over 48 hours. These parameters were then statistically compared at the final time point.

### 2.8. Statistical Analysis

All culture conditions were performed in triplicate for each cell population. All statistical analyses were performed using Analyse-It® software for Microsoft Excel. The Anderson-Darling test for normality was used to assess data. When normally distributed, differences were investigated using Student's *t*-tests whilst Mann-Whitney tests were used for non-normally distributed data. Statistical significance was taken at *p* < 0.05. Data presented are means ± standard error of the mean (SEM).

## 3. Results

### 3.1. Temporal Responses to Nutrient Deprivation

#### 3.1.1. Bovine NP Cell Number

**Figure 2:**
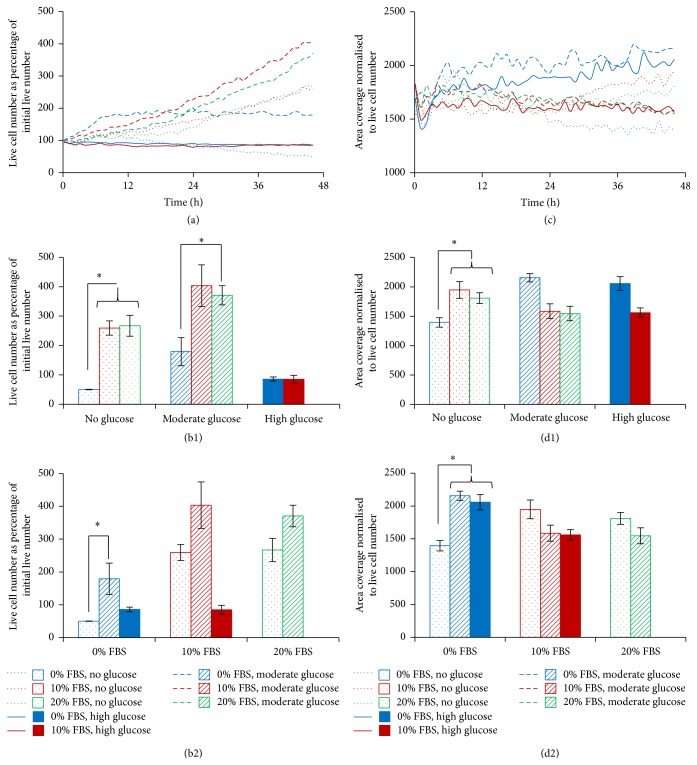
Monitoring the response of bovine NP cell cultures to changes in FBS and glucose concentration over 48 hours. (a) Temporal analysis for live cell number, expressed as the percentage of the initial live cell number. (b) Live cell number, expressed as a percentage of the initial live cell number at the final time point (±SEM), comparing FBS levels within glucose subgroup (b1) and comparing glucose levels within FBS subgroup (b2). (c) Temporal analysis for area coverage normalised to live cell number. (d) Cell area, normalised to live cell number, at the final time point (±SEM), comparing FBS levels within glucose subgroup (d1) and comparing glucose levels within FBS subgroup (d2). *∗* represents a statistically significant difference of *p* < 0.05.

Due to variation in the cell density in each region of interest at the start of the image capture, live cell counts per image were expressed as a percentage of the initial cell count; thus, all cultures began at 100%. Most of the growth conditions tested produced an increase in live cell number over time, except for cultures without FBS or glucose and those with high levels of glucose, which showed a marked reduction in live cell number over the 48 hours, reducing to 50.17, 85.87, and 85.05% (Figure 2(a)).

At the final time point, moderate glucose supplementation to medium containing no FBS produced significantly more live cells than those cultures without FBS or glucose (179.26 versus 50.17%, resp.; *p* < 0.01, Figure 2(b1)). Interestingly, the application of high glucose concentrations seemed to have little effect on live cell number in either the presence or the absence of FBS (85.05 compared to 85.87%, resp.). The addition of FBS to medium containing no glucose significantly enhanced live cell numbers compared to cultures in medium containing no FBS or glucose (259.43 versus 50.17%, resp.; *p* < 0.01). Whilst the addition of 10% or 20% FBS to medium containing moderate glucose produced a greater percentage of live cells than those without FBS, the difference was only significant for 20% FBS (403.59, 370.69, and 179.26%, resp.; *p* = 0.03, Figure 2(b1)).

#### 3.1.2. Bovine NP Cell Area

Over the 48-hour time period assessed, cell area (normalised to live cell number) was reasonably constant (Figure 2(c)). At the final time point, supplementation of glucose to medium containing no FBS produced a significantly larger mean cell area size (1947.53 and 1809.13 compared to 1395.63 *μ*m^2^, resp.; *p* < 0.01, Figure 2(d2)). When the medium contained FBS, the addition of glucose resulted in a reduced cell area (1585.08 compared to 2155.03 *μ*m^2^, resp.), whilst the supplementation of FBS to medium containing no glucose produced a significantly greater cell area compared to those cultures with no FBS or glucose at all (2155.03 versus 1395.63 *μ*m^2^, resp.; *p* < 0.05, Figure 2(d2)). In contrast, when medium contained glucose, the addition of FBS led to a significant reduction in mean cell area (1585.08 versus 1947.53 *μ*m^2^, resp.; *p* < 0.05, Figure 2(d1)).

### 3.2. Human NP Cell and MSC Number

**Figure 3:**
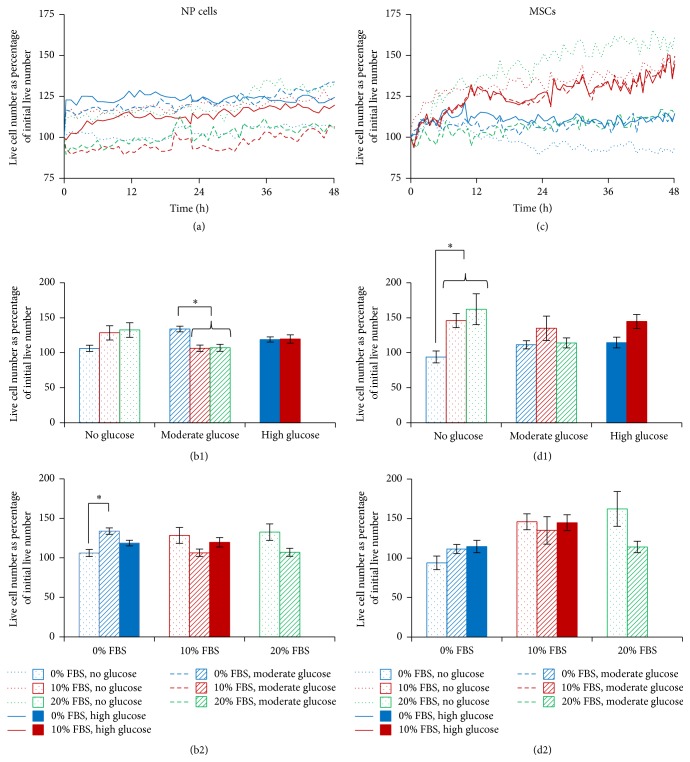
Temporal analysis of number of human NP cells and MSCs in response to altered FBS and glucose concentration, over 48 hours. Trends over 48 hours for human NP cells (a) and human MSCs (c) and at the end time point (±SEM) for human NP cells (b) and human MSCs (d), comparing FBS levels within glucose subgroups ((b1) and (d1)) and comparing glucose levels within FBS subgroups ((b2) and (d2)).

As with bovine NP cells, live cell numbers of human NP cells and MSCs were expressed as a percentage of initial live cell number. Unlike the bovine NP cells, the human NP cells showed less variation in cell number with each condition (Figure 3(a)). However, at the end time point, those NP cells treated with 0% FBS and moderate glucose had a significantly greater number of live cells than those treated with 10 or 20% FBS and moderate glucose (128.44, 106.38, and 119.67%, resp.; Figure 3(b1)) whilst those cells treated without FBS showed significantly greater numbers of live cells when moderate glucose was added, compared to without (128.44 versus 106.14%; Figure 3(b2)). The human MSCs were particularly responsive to altered glucose and showed greater increases in cell number compared to the human NP cells (Figure 3(c)). For the MSCs treated with no glucose, the addition of FBS resulted in significantly more live cells than those without FBS (111.42, 114.55, and 93.89%, resp.; Figure 3(d1)). Further, the concentration of glucose did not significantly alter the live cell number for any of the FBS percentages investigated (Figure 3(d2)).

**Figure 4:**
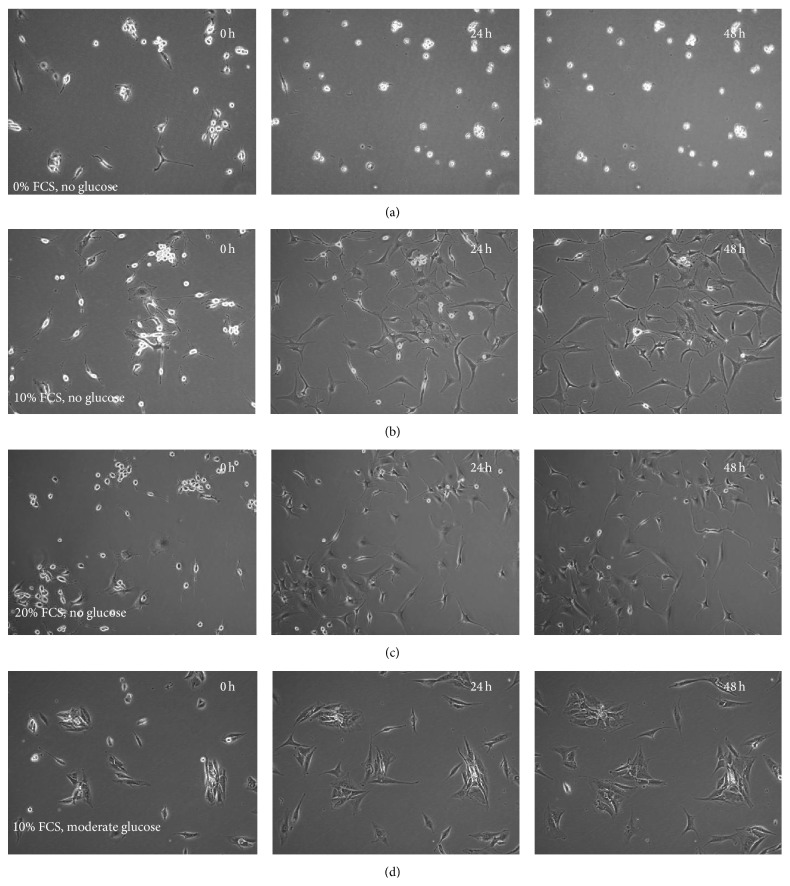
Changes in human NP cellular morphology with time. (a) Cells treated with 0% FBS and no glucose appeared “phase-bright” and semiadhered. This morphology showed no change over time, whereas cells treated with 10% FBS and no glucose (b) and 20% FBS with no glucose (c) showed recovery from a rounded phase-bright morphology to flattened live fibroblast morphology during the 48 hours; (d) cells treated with 10% FBS and moderate glucose were flattened and fibroblast-like and showed no discernible changes in morphology over time.

In all of the conditions without glucose, at least some of the NP cells at initial time points appeared semiadherent, with rounded phase-bright morphologies. These cells we believe are either dividing or dying as mitotic (dividing) and early apoptotic cells both undergo the same morphological changes of becoming rounded and phase-bright. At this point, these cells are alive and will go on to divide or enter apoptosis. Additionally, as observed in the current study, this phase-bright morphology is part of a short-term “shock response” to glucose deprivation. However, as time progressed, in those cultures that had serum supplementation at 10% (Figure 4(b)) or 20% (Figure 4(c)), these semiadhered cells appeared to reattach and returned to a fibroblast-like morphology. If you compare this change in cell morphology to the original sample library (Figure 1(b)), it can be seen how the cells switch categories throughout the time period of the experiment. Cells that had neither serum nor glucose remained in a semiadherent state during the 48 h tested (Figure 4(a)), compared to control cultures supplemented with 10% FBS and moderate glucose (standard NP culture conditions), which maintained a fibroblast-like morphology throughout (Figure 4(d)). No morphological changes like these were seen in the cell populations supplemented with glucose but no FBS.

### 3.3. Human NP Cell and MSC Cell Area

**Figure 5:**
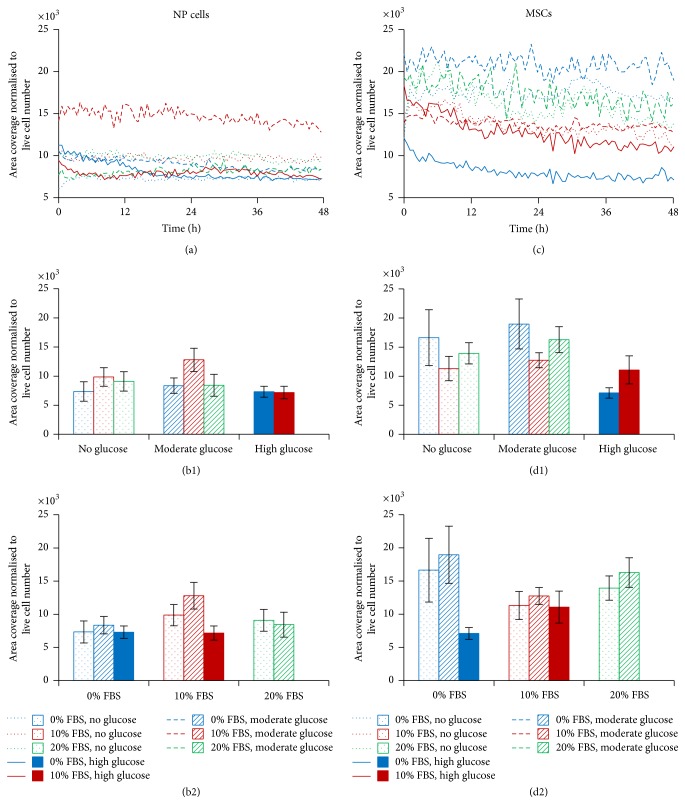
Temporal analysis of human NP cell and MSC area coverage in response to FBS and glucose concentration over 48 hours. Trends over 48 hours for human NP cells (a) and human MSCs (c). Mean area coverage, normalised to live cell number at the final time point (±SEM) for (b) human NP cells and (d) human MSCs, comparing FBS levels within glucose subgroups ((b1) and (d1)) and comparing glucose levels within FBS subgroups ((b2) and (d2)).

The human NP cells showed little change in mean cell area over the 48-hour time period for any condition tested, with the exception of those cells treated with 10% FBS and moderate glucose (Figure 5(a)). These cells remained consistently larger than the others over the 48 hours. At the end time point, the cells treated with 10% FBS and moderate glucose were significantly larger than those without glucose or FBS (12802.7 compared to 7339.4 *μ*m^2^, resp.), whilst no other differences were seen (Figure 5(b)). In contrast, the MSCs, treated with 0% FBS and high glucose, had consistently lower mean cell areas, compared to MSCs grown in other conditions, for the duration of the experiment. Those cells treated with 10% FBS had smaller area coverage than those treated with 20% FBS, whilst the presence of glucose made little difference. Interestingly, the MSCs treated with 10% FBS but no glucose had the largest cell area over the 48-hour experiment (Figure 5(c)). Although these notable differences were mirrored at the end time point, none of these were significant (Figure 5(d)). It is noteworthy that there were no significant differences in the parameters that we analysed (i.e., cell number expressed as a percentage of the starting value or cell area normalised to live cell number) when comparing the initial seeding densities of each culture, that is, 5000 versus 10,000 cells per cm^2^ (data not shown).

### 3.4. Temporal Response of Bovine NP Cells to Induction of Cell Death and Apoptosis

**Figure 6:**
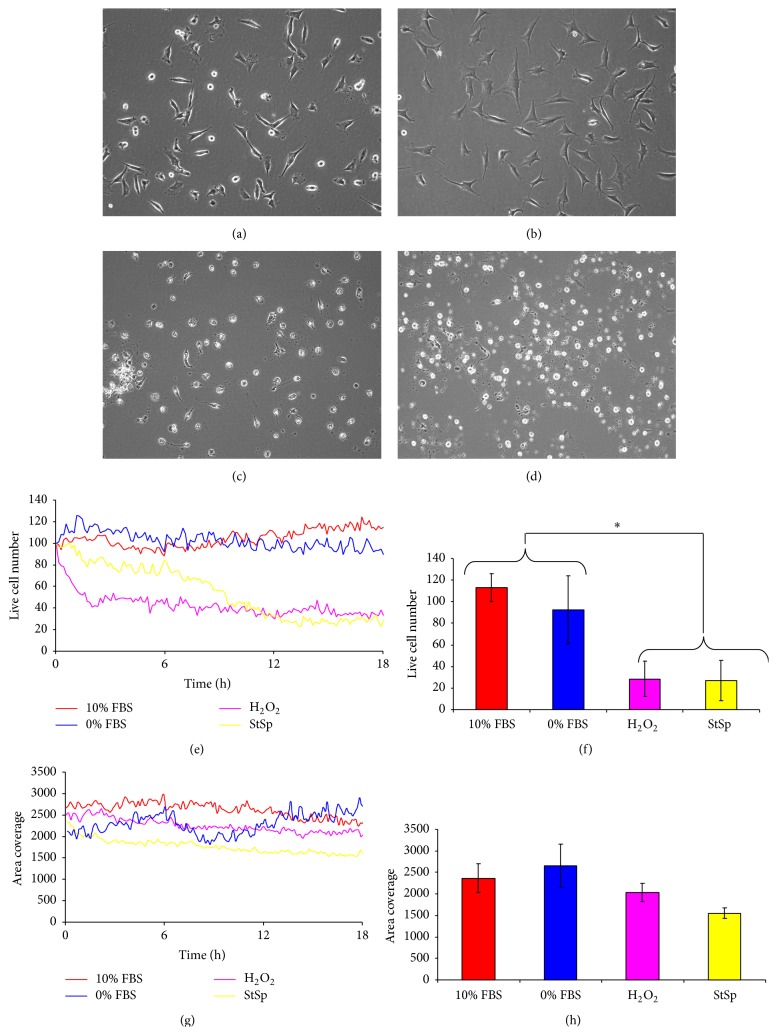
The response of bovine NP cells to induction of cell death inducers over 18 hours. Representative phase contrast images following 18-hour exposure to 10% (a) or 0% (b) FBS with moderate glucose, (c) hydrogen peroxide, and (d) staurosporine. (e) Temporal analysis for live cell number, expressed as percentage of the initial live cell number. (f) Live cell number, expressed as a percentage of the initial live cell number at the final time point (±SEM). (g) Temporal analysis for area coverage normalised to live cell number. (h) Area coverage, normalised to live cell number, at the final time point (±SEM).

NP cells in standard culture conditions (10% FBS and moderate glucose) were used as controls for normal cell growth and compared to those cells treated with inducers of cell death and apoptosis. In control cultures, cells were plastic adherent and fibroblast-like unless they are dividing, in which case cells appeared rounded and phase-bright (Figure 6(a)). Control cell numbers increased modestly over the 18 hours tested, to approximately 110% of the initial number seeded (Figure 6(e)). Generally, FBS-starved cells appeared similarly spread and fibroblast-like, although there was little evidence of cell proliferation; this was reflected in the cell numbers recorded, which remained constant throughout the 18-hour experiment (Figures 6(b) and 6(e)). In contrast, those cultures treated with staurosporine or hydrogen peroxide (H_2_O_2_) contained few, if any, spread or adhered cells; most cells appeared rounded or fragmented at the 18-hour time point (Figures 6(c) and 6(d)) and the live cell number markedly decreased. Cell number of H_2_O_2_ treated cultures decreased more rapidly in the first 3 hours compared to staurosporine treated cells (Figure 6(e)). However, by the final time point, there was no significant difference between H_2_O_2_ or staurosporine treatments (which were 28% and 27% of the initial live cell number, resp.; Figure 6(f)). Both H_2_O_2_ and staurosporine treatments resulted in significantly reduced cell numbers at the 18-hour time point compared to control cultures or those with no FBS (*p* < 0.05; Figure 6(f)).

The mean cell area remained fairly consistent over the 18-hour experiment in all of the conditions tested. Cell areas for cultures without FBS were slightly higher than controls, whereas values for H_2_O_2_ and staurosporine treated cultures were lower than controls. Staurosporine treated cells had significantly smaller cells compared to cells grown under any other condition tested (*p* < 0.05; Figures 6(g) and 6(h)).

## 4. Discussion

Alterations of nutrition in the degenerate IVD may influence a cell therapy approach by dictating which cell type is able to survive and restore disc function. The main aim of this study was to examine the temporal response of NP cells and MSCs (the two main types of cell proposed in the literature for such a procedure) [13–18, 27, 28] to physiologically relevant nutrition depletion* in vitro*. We approached the study in a stepwise manner, first establishing methods (including inducing apoptosis using the “classical” but not physiological stimuli) on bovine NP cells, since this population can be obtained as a more numerous and homogenous population than from humans. We applied “classical” death inducing agents to these, in addition to studying the influence of nutrient depletion, both in human and in bovine cells, but at slightly different time points. The levels of nutrients which the cells were exposed to in these* in vitro* studies were taken from levels that we have used previously and have published in the literature [29]. There is of course some potential limitation here as levels reported for the intervertebral disc have in the main been obtained from either indirect measurements or modelling, since actual direct* in vivo* measurements are limited, particularly in normal human discs, as the methods required to do this are invasive [30].

Firstly, we have shown that human MSCs appear to be more sensitive to nutrient deprivation than human NP cells. In the current study, medium containing no glucose but supplemented with 10% FBS provided up to 0.5 mM of glucose. The critical threshold of glucose concentration for NP cells, at which cell death occurs, is reported to be 0.5 mM [31], thus explaining the survival of NP cells in the low glucose conditions tested in our study. MSCs, however, are derived from a highly vascularised and hence nutrient rich tissue (bone marrow) [32] and may require greater nutrition for survival particularly as blood glucose concentration is 4–5.5 mM [33]. Indeed, other similar immature mesenchymal cells (intervertebral disc notochordal cells) have been reported to require at least 1 mM of glucose for normal metabolism [34]. Further, in agreement with previous studies, we have demonstrated that FBS and glucose starvation had a pronounced effect on bovine NP cell viability, causing a reduction in live cells [5, 35–38]. However, these findings were not echoed in the human NP cell viability, which showed little change when nutrition was altered. We have also included more culture conditions/combinations, for example, high glucose. There are numerous differences between the current study and our previous reported observations [29, 35], the most obvious and significant difference being that in the current study we are also comparing cells isolated from human disc (in addition to bovine) and human bone marrow; the manner in which these human cell populations respond to their environmental conditions is obviously very relevant to their use in developing cellular treatments for disc degeneration and in clinical trials. In addition, we have built on our previous work, when we assessed cells' response to nutrient deprivation at only an end time point [29], by using a very different state-of-the-art methodology (continuous temporal analysis). The live cell imaging techniques used in the current study have made the detailed longitudinal analysis of cellular responses to altered nutrition possible and have shown, for what we believe is the first time, that disc cells show an initial response to changes in levels of FBS and glucose but then are apparently able to adapt. Through these temporal analyses, we have shown that human NP cells undergo an initial loss of adherence in response to glucose deprivation, appearing temporarily rounded and phase-bright, but, in the presence of FBS, recover quickly (within 48 hours) to return to a fibroblast-like morphology, at least in monolayer. These transient observations would otherwise have been missed with end point analysis or would have required intermittent exposure to suboptimal culture conditions for manual image capture and labour intensive quantitation of culture parameters. These potentially important temporal findings are perhaps reflective of the reduced glucose required by NP cells for maintenance of focal adhesions [39], compared to the other cells tested. This suggests the ability of human NP cells not only to survive glucose deprivation, but also to actively recover following exposure to glucose-poor medium. The resilience to reduced nutrition observed in the human NP cultures may reflect the poor environment from which they have originated, that is, the degenerate human IVD, and perhaps is indicative of “hormesis,” where the exposure of cells to mild doses of stressors induces a response that increases stress resistance (i.e., “that which does not kill us makes us stronger”) [40]. Additionally, their recovery may be indicative of NP cells switching to using glycogen stores for an energy source, following the removal of glucose in the culture medium. Indeed, a previous study reported that complete glucose starvation only eliminated lactate production (a commonly used measure of metabolism) after 2 days of exposure [34], possibly following the depletion of glycogen stores which are known to exist in up to 70% of cells from surgically obtained IVDs [24]. Hence, these cells may have developed mechanisms to survive the increasingly harsh environments of the degenerate IVD which may explain their increased ability to withstand a nutritional insult* in vitro* (i.e., no glucose).

Although they are more indifferent to nutrient assault than MSCs, we have demonstrated that NP cells can be induced to undergo cell death, and specifically apoptosis, via H_2_O_2_ and staurosporine application, resulting in rapid NP cell shrinkage, decrease in cell number, and, for staurosporine, cell fragmentation. These changes occurred at a markedly faster rate following H_2_O_2_ treatment compared to staurosporine. It appears that staurosporine and hydrogen peroxide lead to cell death via different mechanisms. Activation of the ERK pathway is the result in cells exposed to hydrogen peroxide [41], whereas staurosporine causes cell death via the release of Cathepsin D from the lysosomes, an event which is independent of oxidative events. In addition, the timescale appears different, with a short insult of one hour's exposure to staurosporine producing cell death over the subsequent 24 hours [41].

We have also attempted to quantitate changes in cell area in response to changes in the amount of FBS and altered levels of glucose. It should be noted that the cell area assessed here is purely a measure of two-dimensional cell spreading and does not take into account any cellular changes in the *z*-axis. Hence, a reduction in cell area may not reflect a reduction in cell volume. Nonetheless, we have shown that the cell area of bovine NP cells was markedly reduced by FBS and glucose deprivation compared to those cells supplemented with any level of FBS or glucose, consistent with the cell shrinkage and phase-bright morphology seen. However, no significant differences in cell area were noted in either human NP or MSC cultures in any of the nutrient conditions assessed. Those reductions in area that have been noted for bovine NP cells may indicate that these cells are in the process of dying in response to nutrient starvation. Alternatively, or perhaps coincidentally, cells may be spreading (indicative of* in vitro* dedifferentiation or hypertrophy) in the presence of FBS or glucose supplementation [42, 43]. Further investigation is required to corroborate these hypotheses. Cell spreading may be indicative of the cells' propensity for adherence to the tissue matrix* in vivo*, although cells may attach differently to the tissue culture plastics* in vitro* than to collagen fibres, for example,* in vivo*.

It is noteworthy that none of the FBS and glucose combinations tested here resulted in a reduction in live cell number in either bovine or human NP cultures or human MSCs in the culture systems studied. However, FBS and glucose starvation was shown to reduce cell proliferation. Previous studies support these findings, reporting that FBS deprivation in NP cultures resulted in reduced cell proliferation and growth arrest, but not an increase in cell death [35]. Indeed, Risbud et al. [44] reported that NP cells actively resist apoptosis following FBS deprivation, via the upregulation of PI3K/Akt and MEK/ERK signalling pathways. Our results appear to show the ability of cells to survive in environments of reduced nutrition, although we have not examined the influence of serum starvation and reduced glucose on alterations in cell phenotype. Omlor et al. [45] showed that reduced levels of glucose resulted in significantly reduced metabolic activity in porcine intervertebral disc cells, whilst reduction of glucose (to 0.45 g/L) in human MSCs has been shown to prevent cellular senescence yet increase proliferative abilities [46]. In other cell types, such as muscle cells, the introduction of high glucose has been shown to inhibit apoptosis normally caused by deprivation of FBS [47]. Further, it has recently been reported that MSCs have an immunomodulatory mechanism of action, via paracrine secretions [48]; therefore, the length of their survival in the nutrient-poor environment of the NP may not need to be long to produce an effect.

We realise that there are limitations to our study, in that we have only assessed cell survival via temporal morphological changes in monolayer culture. The study could have been improved in several ways, for example, by examining how the environment affected the actual functioning of the cells, not only if grown in low nutrient levels but also if grown under hypoxic conditions as can be found in the disc matrix. These further studies would be of benefit and relevance to use of these cells as a therapy for the restoration of the degenerate IVD.

## 5. Conclusions

This study demonstrates that NP cells and MSC react temporally to alterations in their nutrient environment* in vitro*. Specifically, bovine NP cells proliferate less and become smaller in terms of area in response to FBS and glucose deprivation. Human MSC showed similar sensitivity to nutrient deprivation in terms of growth arrest, but not cell area, whilst human NP cells appeared to remain unresponsive to nutrient change in any of the conditions tested or culture parameters analysed. However, there is no evidence that FBS or glucose starvation could initiate cell death in these cultures; in fact, this is only seen following the direct application of toxic stimuli. These findings suggest that human NP cells and MSC could be tolerant to nutrient-poor conditions and might survive if implanted into degenerate IVD tissue. The results of this study may therefore have implications, not only for the understanding of IVD cell biology and pathology, but also perhaps for the development of cell therapy strategies.

## Supplementary Material

Supplementary Table 1: Mean percentage of live cells at the end time point (compared to initial live cell number) for bovine and human NP cultures and human MSCs in each of the defined nutrient conditions tested. Standard error of the mean (SEM) is also noted.
Supplementary Table 2: Mean cell area (*µ*m2) for bovine and human NP cultures and human MSCs in each of the defined nutrient conditions tested. Standard error of the mean (SEM) is also noted.
